# Conservation Genetics of the Philippine Tarsier: Cryptic Genetic Variation Restructures Conservation Priorities for an Island Archipelago Primate

**DOI:** 10.1371/journal.pone.0104340

**Published:** 2014-08-19

**Authors:** Rafe M. Brown, Jennifer A. Weghorst, Karen V. Olson, Mariano R. M. Duya, Anthony J. Barley, Melizar V. Duya, Myron Shekelle, Irene Neri-Arboleda, Jacob A. Esselstyn, Nathaniel J. Dominy, Perry S. Ong, Gillian L. Moritz, Adrian Luczon, Mae Lowe L. Diesmos, Arvin C. Diesmos, Cameron D. Siler

**Affiliations:** 1 Biodiversity Institute and Department of Ecology and Evolutionary Biology, University of Kansas, Lawrence, Kansas, United States of America; 2 Biodiversity Institute, University of Kansas, Lawrence, Kansas, United States of America; 3 Institute of Biology, University of the Philippines Diliman, Quezon City, Philippines; 4 Diliman Science Research Foundation Inc., University of the Philippines Diliman, Quezon City, Philippines; 5 Research Institute of EcoScience, Ewha Womans University, Seoul, Republic of Korea; 6 Thomas Glen Erin Animal Hospital, Mississauga, Ontario, Canada; 7 Museum of Natural Science & Department of Biological Sciences, Louisiana State University, Baton Rouge, Louisiana, United States of America; 8 Department of Anthropology, Dartmouth College, Hanover, New Hampshire, United States of America; 9 Institute of Biology, University of the Philippines, Diliman, Quezon City, Philippines; 10 Department of Biological Sciences, Dartmouth College, Hanover, New Hampshire, United States of America; 11 University of Santo Tomas, Espana, Manila, Philippines; 12 Herpetology Section, Zoology Division, Philippine National Museum, Ermita, Manila, Philippines; 13 Sam Noble Oklahoma Museum of Natural History and Department of Biology, University of Oklahoma, Norman, Oklahoma, United States of America; Macquarie University, Australia

## Abstract

Establishment of conservation priorities for primates is a particular concern in the island archipelagos of Southeast Asia, where rates of habitat destruction are among the highest in the world. Conservation programs require knowledge of taxonomic diversity to ensure success. The Philippine tarsier is a flagship species that promotes environmental awareness and a thriving ecotourism economy in the Philippines. However, assessment of its conservation status has been impeded by taxonomic uncertainty, a paucity of field studies, and a lack of vouchered specimens and genetic samples available for study in biodiversity repositories. Consequently, conservation priorities are unclear. In this study we use mitochondrial and nuclear DNA to empirically infer geographic partitioning of genetic variation and to identify evolutionarily distinct lineages for conservation action. The distribution of Philippine tarsier genetic diversity is neither congruent with expectations based on biogeographical patterns documented in other Philippine vertebrates, nor does it agree with the most recent Philippine tarsier taxonomic arrangement. We identify three principal evolutionary lineages that do not correspond to the currently recognized subspecies, highlight the discovery of a novel cryptic and range-restricted subcenter of genetic variation in an unanticipated part of the archipelago, and identify additional geographically structured genetic variation that should be the focus of future studies and conservation action. Conservation of this flagship species necessitates establishment of protected areas and targeted conservation programs within the range of each genetically distinct variant of the Philippine tarsier.

## Introduction

Biodiversity-rich tropical forests are being degraded worldwide, and the pace of forest destruction is exceptionally rapid in insular Southeast Asia [1]. With only 4–8% of its original forest remaining [2], the Philippines has been designated as both a global conservation biodiversity hotspot [3] and a Megadiverse nation [4]—a distinction shared only with Madagascar. Within this archipelago, the Philippine tarsier, a small endemic nocturnal primate, has been enlisted as a flagship species for an emerging societal conservation movement and an expanding ecotourism industry [1], [5].

Traditionally, taxonomy and conservation have been inextricably linked and most conservation strategies have targeted formally named taxonomic units: species or subspecies [6]. Although most conservation efforts have targeted these taxonomic entities, conserving finer-grained genetic diversity across a species' range [7], [8] is essential to preserving metapopulation dynamics, preventing inbreeding depression, avoiding population collapses, and ultimately ensuring against extinction [9]–[11]. Unique evolutionary lineages or genetically defined “Evolutionarily Significant Units” [12] are appropriate targets of conservation programs aimed at preserving genetic diversity among and within species; targeting empirically defined distinct evolutionary lineages has the added benefit of potentially removing the subjectivity sometimes associated with traditional taxonomy [13].

**Figure 1:**
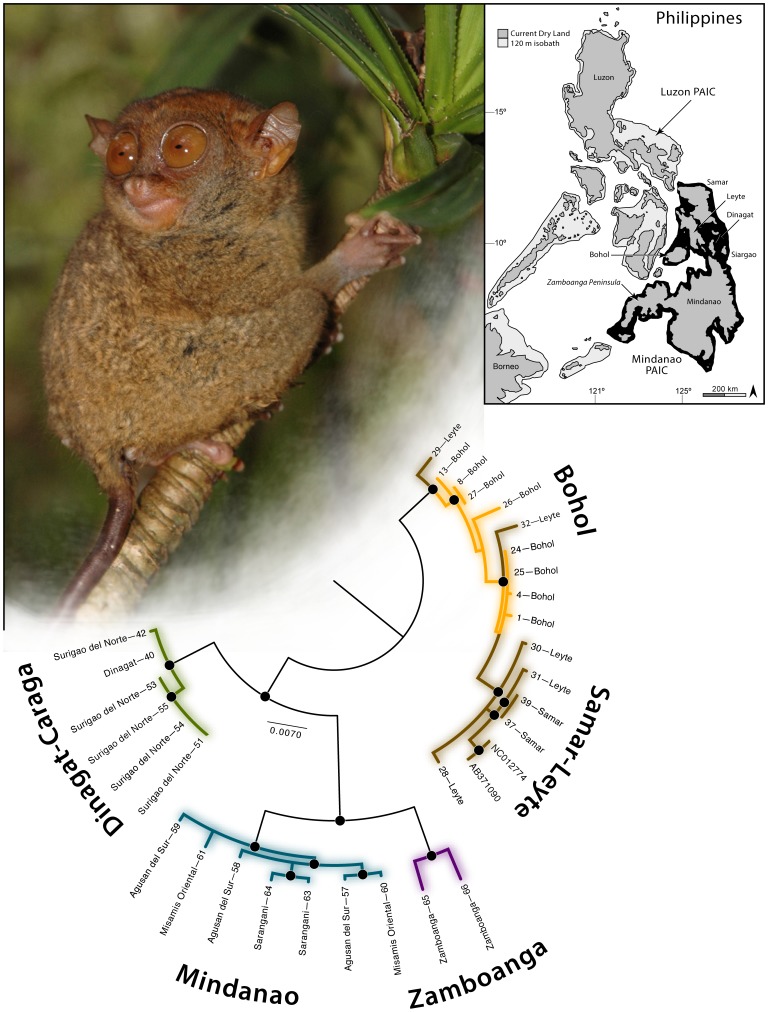
Phylogeographic relationships of *Tarsius syrichta* (see Appendix S1 for taxonomic summary) estimated from a combined, partitioned, RAxML ML analysis of mitochondrial (12S, CytB, ND2) gene fragments. Black circles at nodes correspond to ML bootstraps ≥70% and Bayesian PP ≥95%.

Despite being the focus of a disproportionate number of intensely focused studies, multiple lines of evidence suggest that numerous primate taxa await discovery and formal taxonomic description. For example, between the 1975 and 1999 nocturnal primate species diversity grew worldwide by 2.85-fold increase [14]–[18], and has since climbed by an additional 1.69-fold increase [19]. Unique among nocturnal primates, tarsiers are found only in insular Southeast Asia (See Appendix S1). Ten species are recognized currently, with several new taxa recently proposed through bioacoustic analysis and molecular data [15]. The Philippine tarsier (Fig. 1) has been the focus of recent attempts to understand morphological variation [16], clarify taxonomy [17], and establish the conservation status of populations [18], [19]; these efforts have met with limited success and left unanswered the questions of appropriate targets for conservation action.

In the Philippines, many morphologically indistinguishable yet genetically differentiated “cryptic” species [20]–[22] have recently been discovered, suggesting that the codistributed Philippine tarsier might also harbor hidden evolutionary lineages (unrecognized species, genetic variants, putative taxa, etc.), which may be unprotected and not incorporated into current conservation planning.

We assessed the conservation genetics of Philippine tarsier to determine (1) how many distinct evolutionary lineages can be identified, (2) whether genetic structure conforms to biogeographical predictions and/or (3) expectations derived from current taxonomy (three subspecies). Due to limited natural history data or consensus from other sources of information (morphology, bioacoustics, ecology), we argue that genetic data should be used to distinguish evolutionarily distinct tarsier population groups as objective, empirically defined conservation priorities.

## Materials and Methods

### Mitochondrial data collection

**Figure 2:**
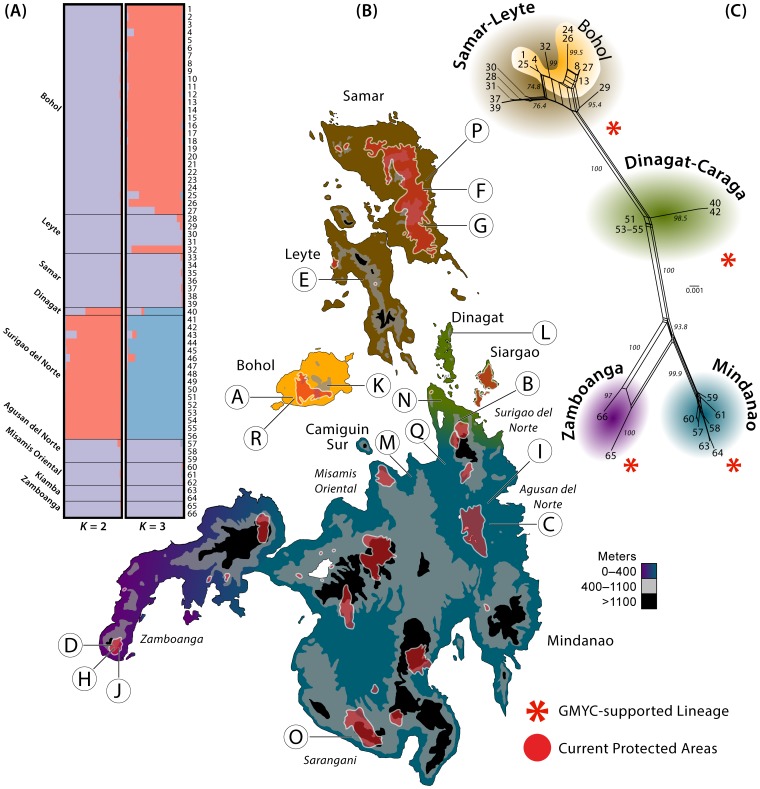
DISTRUCT visualization of STRUCTURE analyses (A) assigning individuals to major population groupings (genetically distinct evolutionary lineages) for Philippine tarsier demes (*K* = 2 and 3 populations). Mindanao faunal region (B; see Fig. 1, inset) with sampling (17 sites, 66 individuals) labeled with letters corresponding to full localities listed in Appendix S2, protected areas shaded red. SplitsTree gene network (C; numbers at internodes = ML bootstrap replicates), and results of GMYC analyses (red asterisks denote lineages delineated by the Yule-coalescent), with numbers at tips corresponding to individual samples in Structure plots (A) and cluster shading corresponding to islands on map (B).

Genetic material from *T. syrichta* was nondestructively sampled (ear and tail-tip biopsies) from throughout as much of the species' range as feasible (targeting ranges of all described subspecies; Appendix S1) on the large islands of Mindanao, Samar, Leyte, Bohol, and Dinagat, Philippines (Figs. 1, 2; Appendix S2). Deceased animals were salvaged from illegal roadside animal dealers, local Provincial or City Environmental Natural Resources (PENRO, CENRO) officers or university administrators (confiscations of poached animals by students, animal traders, and bush meat hunters), and from indigenous hunters in forested areas. Salvaged animals were prepared as museum specimens and deposited at the Leyte State University (ViSCA) Natural History Museum, the National Museum of the Philippines, or the University of Kansas Biodiversity Institute (Appendix S2). We sampled 77 individuals of *T. syrichta* from 17 localities for genetic material from most of the large islands within the Mindanao Pleistocene Aggregate Island Complex (PAIC) [2], including representatives of all recognized subspecies. We sequenced the mitochondrial 12S ribosomal RNA (12S), Cytochrome B (CytB), and NADH Dehydrogenase Subunit 2 (ND2) gene fragments and nine nuclear microsatellites.

Genomic DNA was extracted from tissues following Fujita's guanidine thiocyanate method [23]. Molecular data were derived from all available genetic samples and included efforts to determine the sequence of the 12S mitochondrial gene fragment, the ND2, and the CytB gene region. Sequence identity was confirmed via examination of amino acid translations for internal stop codons for Cytb and ND2 sequence alignments, GenBank BLAST searches, and alignment comparisons with annotated, published comparative sequence data available on GenBank. All sequences were deposited in GenBank under accession numbers KM217270–99 (12S), KM217300–23 (CytB), and KM217324–49 ND2 (Appendix S2). Primers used for amplifying mitochondrial gene sequences include: 12S (Tars12s_AJB.F1, 5′–TCACAACGTCTTGCTCAACC–3′ and Tars12s_AJB.R2, 5′–TTGAGGAGGGTGACGGGCGG–3′); ND2 (TarND2_AJB.f2, 5′–ACTTTCTAATTCAAGCGACAGCCTCC–3′ and TarND2_AJB.r2, 5′–TGGGGGATATGGGTAAAAGTAGGGTGG–3′); CytB (TarsCytB.F1, 5′–CACATCTGCCGAGACGTAAA–3′ and TarsCytB.R1, 5′–TGGGGTGGAGTGTTTAGAGG–3′). We used the following thermal profiles for the mitochondrial fragment amplification: 1 min at 95°C followed by 38 cycles of 94°C for 30 sec, 30 sec at the primer specific annealing temperature (60°C for 12 s; 61°C for ND2; 62°C for CytB), and 72°C for 1 min, and a final extension phase at 72°C for 10 min.

Amplified products were visualized on 1.5% agarose gels and PCR products were purified with 1 µL of a 20% dilution of ExoSAP-IT (US78201, Amersham Biosciences, Piscataway, NJ) on the following thermal profile: 30 min at 37°, followed by 15 min at 80°. Cycle sequencing reactions were run using ABI Prism BigDye Terminator chemistry (Ver. 3.1; Applied Biosystems, Foster City, CA), and purified with Sephadex Medium (NC9406038, Amersham Biosciences, Piscataway, NJ) in Centri-Sep 96 spin plates (CS-961, Princeton Separations, Princeton, NJ). Purified products were analyzed using an ABI Prism 3130xl Genetic Analyzer (Applied Biosystems). Sequence contigs were assembled and edited using Sequencher 4.8 (Gene Codes Corp., Ann Arbor, MI).

Sequencing proved highly problematic for our many degraded tissue samples and approximately half our samples would not amplify for any targeted gene regions. Of the half that could be amplified for >2 target regions, initial sequence alignments were produced in Muscle [24] and manual adjustments were made in MacClade 4.08 [25]. We estimated the phylogeny for each mitochondrial gene fragment independently using likelihood and concatenated mitochondrial sequences following no observation of statistically significant incongruence between datasets. Exploratory analyses of the combined dataset of 36 individuals inferred relationships that did not strongly conflict with topologies inferred from individual gene fragments.

### Microsatellite data collection

We sampled a total of 66 individuals from 17 localities of *Tarsius syrichta* across the southern Philippines. Each locality was represented by 1–27 individuals (mean = 3.88). DNA was extracted using QIAGEN DNeasy extraction kits and we used nine microsatellite makers previously developed for *Tarsius syrichta*: T5, T6, T22, T34, T35, T43, T69 [26] and *Tarsius* spp.: T42, T50 [27]. PCR reaction volumes and conditions for the PCR amplifications followed the protocol of Schuelke [28]. Conditions of the PCR amplification were as follows: 94°C for 30 s, 56°C for 45 s, 72°C for 45 s, followed by 8 cycles at 94°C for 30 s, 53°C for 45 s, 72°C for 45 s, and a final extension at 72°C for 10 min. Primers T42, T43 and T45 were fluorescently labeled with 6-FAM by Integrated DNA Technologies (Coralville, Iowa) for PCR. Primer annealing temperatures for these three loci were as follows: T42 at 53°C [27], T43 at 54°C and T45 at 57°C [26]. Subsequently, 2.1 µl of the PCR product was added to 9 µl of formimide and .15 µl was electrophoresed with the LIZ-500 size standard and analyzed on a 3730 Genetic Analyzer (Applied Biosystems). Fragments were sized with GeneMapper version 4.1 (Applied Biosystems). We replicated data collection for all samples a minimum of two times per locus in order to monitor for allelic dropout and false alleles. In cases where we detected allelic dropout or inconsistent genotypes (n = 2), the sample was replicated once more in order to minimize genotyping errors. In a few instances of ambiguous genotypes, despite these efforts, samples were simply excluded from subsequent analyses (n = 8). Microsatellite data were archived at Dryad (doi:10.5061/dryad.r7468).

### Phylogenetic analyses and lineage delimitation: mitochondrial data

Partitioned Bayesian analyses of the combined dataset were conducted in MrBayes v3.1.2 [29]. As the mtDNA genes sampled in this study included incomplete sections for some targeted genes, datasets were partitioned by gene only. The Akaike Information Criterion (AIC) implemented in jModeltest [30] was used to select the model of nucleotide substitution for each subset (Table 1). We ran four independent Metropolis-coupled MCMC analyses, each with four chains and the default heating scheme (temp = 0.2). All analyses were run for 20 million generations, sampling every 1000 generations. To assess stationarity, all sampled log-likelihoods and parameter values from the cold Markov chain were plotted against generation time and compared among independent runs using Tracer v1.4 [31]. These runs demonstrated patterns consistent with stationarity after 4 million generations, hence the first 20% of samples were discarded as burn-in.

**Table 1:** Models of evolution selected by AIC and applied for partitioned, phylogeographic analyses^1^.

Partition	AIC Model	Number of Characters
12S	GTR + Γ	618
Cytb	HKY + Γ	557
ND2	HKY + Γ	538

1 The model GTR + Γ was used for partitioned RAxMLHPC analyses.

Partitioned maximum likelihood (ML) analyses were conducted in RAxMLHPC v7.0 [32] for the combined dataset, using the same partitioning strategy as implemented in the Bayesian analyses. The more complex model (GTR + I + Γ) was used for all subsets, and 100 replicate ML inferences were performed for each run. Each inference was initiated with a random starting tree, employed the rapid hill-climbing algorithm and support was assessed with 1000 bootstraps [33].

To generate an ultrametric phylogeny for our Generalized Mixed Yule-Coalescent (GMYC) analysis, we ran a strict clock divergence time analysis of the mtDNA data, partitioning the data by gene, and fixing the mean substitution rate to 1.0 in BEAST v1.7.5 [34]. We first removed all individuals with identical sequences, however, since zero-length branches can cause the GMYC analysis to over-partition the dataset. BEAST analyses were run for 100 million generations, sampling every 10,000. Convergence was assessed with Tracer as described above. We then used the ultrametric phylogeny with the “single threshold” GMYC model [35] to determine the number of evolutionary lineages in the mtDNA dataset.

### Population structure and gene flow: nuclear data

As currently recognized, *Tarsius syrichta* spans the Mindanao PAIC in the southern Philippines. As an initial starting point, our *a priori* hypothesis of population structure, given our understanding that all these islands are part of the same faunal region [2], were that genetic diversity might be partitioned among individual islands. In order to test these expectations, we used the Bayesian clustering method of the program Structure v2.3.3 [27], [28], [36]–[39] with allelic data from nine nuclear loci, to estimate the number of populations among *T. syrichta* samples, as well as infer the probabilities of individuals belonging to each of the estimated populations. We considered individuals with *q* values between 0.10 and 0.90 to be admixed [39]–[41]. Due to the absence of *a priori* knowledge of inter-locus relationships, analyses were run under the correlated allele frequency model and the admixture ancestry model [36], [40]. All other parameters were left with default settings. Taking a conservative approach to evaluating population boundaries for this study, we considered the number of possible populations, represented by *K*, to range from one (a single geographically non-structured species) to seven (each island sampled in this study, and allowing for additional population genetic complexity that may be present on the large island of Mindanao [2]). We conducted 10 independent runs in Structure for each of the seven values of *K* (1–7). We ran each analysis for 10 million iterations, with a burn-in of 500,000. The rate of change of *K* (ΔK) was evaluated in Structure Harvester (Web v0.6.93) [42] following the method of Evanno et al. [43] to determine the number of preferred genetic clusters. The results of structure analyses were visualized using the program *Distruct* v1.1 (Fig. 2) [44].

To assess whether there is evidence of recent migration among populations, we estimated the level of historical gene flow within a Bayesian framework in the program BAYESASS v1.3 [45] for the four evolutionary lineages supported in phylogenetic analyses (Fig. 1). Four independent runs were conducted, each with 20,000,000 iterations, a sampling frequency of 2,000, and a burn-in of 2,000. Trace files were assessed using the program Tracer v1.5 [31] for evidence of convergence prior to result summary.

To visualize population genetic structure, we generated phylogenetic networks for the mitochondrial dataset by employing the Neighbor-Net algorithm [46] in the program SplitsTree v4.10 [47] (Fig. 2). To assess the support for inferred splits in the network, a bootstrap analysis was conducted with 1000 pseudoreplicates.

## Results

### Genetic variation and gene networks

Of the 77 samples collected, 66 could be characterized for microsatellite variation (Tables 2, 3) but, due to sample degradation, only 36 could be sequenced for mitochondrial DNA. As anticipated, the results of network analyses corroborate the major results observed from phylogenetic analyses (Figs. 1, 2); these same mitochondrial clades were identified as distinct in the GMYC lineage delineation procedure.

**Table 2:** Microsatellite variation in 66 individuals of *Tarsius syrichta* from 17 localities (Fig. 2).

Population	*N*	*H*_O_	*H*_E_	*L*_P_	*F*_IS_	*P* value
Bohol	27	0.58674±0.27641	0.74946±0.16146	9	−0.12254	0.859238
Samar-Leyte	12	0.53519±0.24444	0.80535±0.07257	6	0.10995	0.214076
Dinagat-Caraga	17	0.53184±0.19296	0.63060±0.12853	4	0.0764	0.304008
E. Mindanao	8	0.63294±0.17635	0.83488±0.12227	7	0.48611	0.008798
W. Mindanao	2	0.56250±0.32043	0.75000±0.17817	8	0.42857	0.339198

Abbreviations include: *N*, number of samples; *H*_O,_ observed heterozygosity; *H*_E,_ expected heterozygosity; *L*_P_, number of polymorphic loci; *F*_IS_ inbreeding coefficient.

**Table 3:** Locus-specific microsatellite variation in 66 individuals of *Tarsius syrichta* from 17 localities (Fig. 2).

		Bohol	Samar-Leyte	Dinagat-Caraga	E. Mindanao	W. Mindanao
Locus T22	*H_O_*	0.62963	0.75	0.64286	0.5	0.5
	*H_E_*	0.76799	0.80435	0.5291	0.83333	0.83333
	*A_N_*	6	9	6	6	3
	*HWE*	**0.0064**	0.335	1	0.0562	0.3301
Locus T35	*H_O_*	0.69231	0.75	0.6875	0.5	n/a
	*H_E_*	0.7911	0.7971	0.68347	0.53333	n/a
	*A_N_*	8	8	5	5	n/a
	*HWE*	**0.0211**	0.7219	0.4304	0.279	n/a
Locus T5	*H_O_*	0.84615	0.58333	0.41176	0.375	0.5
	*H_E_*	0.89819	0.84783	0.58824	0.84167	0.5
	*A_N_*	10	9	5	5	2
	*HWE*	0.2585	**0.0039**	0.1024	**0.0046**	n/a
Locus T6	*H_O_*	0.46154	0.08333	0.35294	0.5	0
	*H_E_*	0.79864	0.82246	0.67023	0.93333	0.66667
	*A_N_*	11	7	7	9	2
	*HWE*	**0.0**	**0.0**	**0.0134**	**0.0**	0.3346
Locus T34	*H_O_*	0.57692	0.4	0.23529	0.875	0.5
	*H_E_*	0.73454	0.73684	0.36898	0.925	0.83333
	*A_N_*	6	5	5	10	3
	*HWE*	**0.0161**	**0.0204**	**0.0245**	0.1925	0.3305
Locus T42	*H_O_*	0.85185	0.66667	0.53846	0.85714	0.5
	*H_E_*	0.8833	0.86928	0.77231	0.89011	0.5
	*A_N_*	11	8	5	8	2
	*HWE*	**0.0**	**0.0064**	0.4807	0.4357	1
Locus T69	*H_O_*	0.18519	0.33333	0.45455	0.75	1
	*H_E_*	0.53319	0.66013	0.58009	0.79167	0.83333
	*A_N_*	5	4	6	5	3
	*HWE*	**0.0009**	0.0862	**0.048**	0.2565	n/a
Locus T43	*H_O_*	0.88889	0.83333	0.875	0.625	0.5
	*H_E_*	0.89727	0.9058	0.7379	0.90833	0.83333
	*A_N_*	13	11	6	9	3
	*HWE*	0.562	0.0843	0.1855	**0.0**	0.3384
Locus T45	*H_O_*	0.14815	0.41667	0.58824	0.71429	1
	*H_E_*	0.44095	0.80435	0.7451	0.85714	1
	*A_N_*	6	7	8	6	4
	*HWE*	**0.0**	**0.0037**	0.0542	0.1938	1

Abbreviations include: *H*_O,_ observed heterozygosity; *H*_E,_ expected heterozygosity; *A_N_*, number of alleles; *HWE*, deviation from Hardy–Weinberg equilibrium (p-value).

### Phylogenetic and lineage delimitation analyses

GMYC analysis of the mitochondrial phylogeny identified three putative evolutionary lineages; each of these three lineages was supported in Bayesian and ML phylogeographic analyses and phylogenetic networks of mitochondrial sequence data, and were provisionally adopted as the following distinct evolutionary groups (Fig. 1): (1) Bohol, Samar, and Leyte island, (2) Dinagat and Northeast Mindanao (Caraga Region) island, and (3) Mindanao island, composed of (*a*) eastern and (*b*) western subclades; substantial genetic divergence was found among groups (2.1–4.7% in mtDNA; Table 4), with markedly less genetic variation within groups (0.0–1.1%) and minimal gene flow between most groups (Table 5).

**Table 4:** Uncorrected mitochondrial sequence divergence (%) among Philippine tarsier (*Tarsius syrichta*) evolutionary lineages shown below diagonal.

	Bohol	Samar-Leyte	Dinagat-Caraga	E. Mindanao	W. Mindanao
**Bohol**	0.0–0.9	0.018	0.015	0.018	0.026
**Samar-Leyte**	0.4–1.4	0.0–1.1	0.019	**0.065**	**0.056**
**Dinagat-Caraga**	2.1–3.5	2.1–3.4	0.0–2.3	0.021	0.029
**E. Mindanao**	3.4–4.6	3.5–4.6	2.7–4.6	0.1–0.8	**0.051**
**W. Mindanao**	3.8–4.6	3.7–4.6	3.0–4.7	2.0–2.4	0.0–1.0

Percentages on the diagonal represent intraspecific (or within clade) genetic diversity. Mean inferred migration rates (inferred in BAYESASS) between regionally partitioned diversity shown above diagonal. Migration rates above the 0.05 threshold bolded for emphasis.

**Table 5:** Identities by geographical region and numbers (in parentheses) of Philippine tarsier (*Tarsius syrichta*) evolutionary lineages, putative taxa, or conservation targets inferred (new data and analyses) or predicted (biogeography, taxonomy) from each of the five available sources of information.

Divergent mtDNA lineages & GMYC results (4)	Distinct nuDNA groups (3)	Islands (5)	Taxonomy (3)
Bohol-Samar-Leyte	Dinagat-Caraga	Bohol	Bohol
Dinagat-Caraga	Bohol- Samar-Leyte-east Mindanao-west Mindanao	Samar	Samar-Leyte
Mindanao (a: east Mindanao, b: west Mindanao)		Leyte	Caraga-Mindanao-Zamboanga
		Dinagat	
		Mindanao	

### Assignment of individuals to population clusters

The *ad hoc* Δ*K* test of the results of Structure analyses of microsatellite loci preferred a *K* of two (LnP[*K*] = −2341.58; Dinagat-Caraga vs. all remaining samples), suggesting an initial estimate of only two distinct populations as defined by our nuclear data alone. All individuals were assigned to one of these two genetic clusters with *q* values above 0.90. With the exception of populations from Dinagat Island and the northeastern peninsula of Mindanao Island (Surigao del Norte Province; Fig. 2), all sampled populations were inferred to be part of one cohesive genetic group. Evaluating a three-population model in structure reveals a similar pattern of unambiguous individual assignments to populations; however, the model of *K* = 3 which was close to maximally preferred included additional support for the distinctiveness of populations on Bohol (Fig. 2). All Structure analyses provided support for the cryptic Dinagat-Caraga population (Fig. 2).

## Discussion

### New Philippine tarsier evolutionary lineages: novel conservation priorities

The unambiguous support across all analyses for a genetically distinct Dinagat-Caraga tarsier lineage, mirrored in all analyses of all loci, is novel, and identifies a range-restricted “cryptic” evolutionary entity with unique conservation concerns. Although moderately forested, Dinagat and northeast Mindanao are impoverished economically, lack low-elevation protected areas (Fig. 2), and have become the focus of particularly intensive mining operations—all of which threaten the remaining suitable habitat of this newly documented evolutionary lineage. The Dinagat-Caraga tarsier should therefore be regarded as tantamount to the conservation importance of celebrated Philippine flagship species (e.g., Philippine eagle, tamaraw, golden-spotted monitor lizard, etc.: [2]). Nearby Siargao Island is protected and may harbor the same genetic variant identified here from Dinagat; intensive studies of these populations are urgently needed.

We anticipate future studies will further refine the known distribution of the Dinagat-Caraga tarsier and extend its range to nearby Siargao Island. Additionally it is not clear if the Surigao del Norte (NE Mainland Mindanao, Caraga Region) samples identified here occur naturally on Mindanao or are the result of Dinagat and/or Siargao island animals confiscated from smugglers by regional wildlife enforcement and subsequently released on Mindanao (PSO, *personal communication* with Surigao del Norte Provincial Environmental Natural Resources Office [PENRO] staff).

### Geographical distribution of genetic variation

Our empirical findings are more complex than is reflected in current taxonomy [18], [48], stand in contrast to past biogeographic paradigms [2], [49], uncover a novel conservation target (Dinagat-Caraga lineage; Figs. 1, 2), and identify a conservation research priority of urgent concern (potential differentiation between western and eastern Mindanao). The moderate level of sequence divergence (2.4%; Table 4) detected between eastern and western Mindanao (both supported as distinct lineages in the GMYC analysis, but with marginally significant migration rates detected in analysis of microsatellite data; Table 4) may indicate that separate tarsier conservation programs for these populations are warranted if future studies confirm their distinctiveness. An east–west sampling transect across Mindanao will be necessary to investigate the genetic relationships of these populations which, given the gap in our sampling, may simply be the extremes of natural geographically based genetic structure.

Our results from analyses of mitochondrial data do not differentiate the populations on Bohol from those on Samar-Leyte (currently recognized as separate subspecies: [48]). Considering the suboptimal *K* = 3 results, Structure analyses of microsatellites identified Bohol potentially as distinct. However, given that these analyses potentially are sensitive to unequal sampling, and that we possessed many more samples from Bohol than from Samar-Leyte, we suspect this result may be an artifact; future studies with better sampling from Samar and Leyte islands, and additional microsatellite loci will be necessary to investigate further genetic relations within the Bohol-Samar-Leyte clade. At present, in our efforts to adhere to objective, empirically defined evolutionary lineages, we stop short of defining Bohol as a distinct conservation target, and point to the nested nature of the Bohol samples within the Bohol-Samar-Leyte tarsier group (Figs. 1, 2).

### Combining mitochondrial and nuclear gene signals to establish targets for conservation action

Under current implementation of international conservation status assessment (IUCN, 2014), prioritization of populations for conservation action follows the recognition of named taxonomic units, i.e., species and subspecies. In the face of differing taxonomic arrangements, biogeographic expectations, and differing geographic patterns of nuclear versus mitochondrial genetic variation, how should the Philippine tarsier be prioritized for implementation of applied conservation measures? Currently, one tarsier sanctuary has been established on Bohol Island and another is under consideration for construction on Leyte. Would these two, and only these two efforts adequately conserve genetic components of Philippine tarsier diversity? We argue that they would not. Would establishment of conservation programs based on current tarsier taxonomy (one effort on Bohol, another on Samar-Leyte, and a third on Mindanao) properly conserve the genetic variation we have documented? Similarly, we argue that such an approach would fail to conserve the genetic variation elucidated here.

Brandon-Jones et al. [17] characterized the three subspecies (one from Bohol, another from Leyte-Samar, and a third from Mindanao) as “dubious” taxa. They wrote: “Groves (2001) recognized no subspecies for *Tarsius syrichta*. Hill (1955) recognized *T. s. syrichta/carbonarius/fraterculus* as a poorly defined subspecies, perhaps synonymous, with *T. s. syrichta*. Museum specimen variation seemed insignificant to Niemitz (1984), but an inadequate basis for judgment, according to Musser, and Dagosto (1987).” Our study sheds some light on the uncertainty of Brandon-Jones et al. [17] and suggests that a detailed study employing other lines of evidence is still needed to revise Philippine tarsier taxonomy. One possible outcome of further study could be that our evolutionary groups are not sufficiently distinct based on new lines of evidence (morphology, bioacoustics, whole genome data) to warrant taxonomic separation, and that Philippine tarsiers should therefore be classified as a single taxon, *T. syrichta* [16], [50], [51]. Alternatively, further investigation might find two or more of our conservation priority groups warrant taxonomic separation, in which case, the epithets applied by Hill [52] will be available (as species or subspecies). Three names are available for a subset of the evolutionary lineages we identify: (1) Samar-Leyte, (2) Bohol, and (3) eastern Mindanao (but not including western Mindanao [Zamboanga], or the novel Dinagat-Caraga lineage). For the immediate future, we argue that applied conservation efforts based upon the combined intersection of our variable genetic results are superior to those based upon existing taxonomy.

A number of genetic and social system phenomena could conceivably account for the differences in lineage-specific support we have inferred between mitochondrial and nuclear loci. Possibilities include persistence of genetic polymorphism, lineage sorting, nuclear gene flow between distinct mitochondrial lineages, sex-biased dispersal, mitochondrial gene sweeps, or an undocumented Philippine tarsier mating system. Given the present sampling, we are unable to distinguish between these possibilities, which may provide intriguing questions for future research. However, from a practical and applied conservation perspective, we argue that a single, clearly optimal, and objective solution results from the combination of patterns observed here in mitochondrial and nuclear gene loci.

Given our variable inferences, we advocate a combined, cumulative approach towards identification and recognition of tarsier evolutionary lineages for conservation planning. Thus, we argue that in order to maximally preserve genetic variation, it is the *combined* results of our variable sources of information that likely will have the best chance of maximally preserving genetic variation in the Philippine tarsier. As such, we advocate a multi-tiered conservation program involving conservation programs and protected areas in the ranges of each distinctive population lineages identified here, minimally including (1) Bohol-Samar-Leyte, (2) Dinagat-Caraga, and (3) Mindanao. Until a comprehensive taxonomic study using multiple lines of evidence (molecular, morphological and/or bioacoustic data) can be undertaken, we find that the existing evidence provides an inadequate basis for distinguishing between taxonomic alternatives. Instead, we emphasize that the archipelago's tarsier populations are partitioned into at least the three genetic variants, which we have empirically defined here for practical conservation purposes. We caution primatologists from taking taxonomic action until multiple lines of evidence converge on a meaningful solution [20], [22], ideally with information from throughout the genome [53] and more robust sampling along multiple transects from throughout the range of this species (i.e., eastern versus western Mindanao). Nevertheless, our results necessitate a refined conservation strategy in order to effectively preserve the geographical distribution of genetic variation in this flagship species. Such an approach will greatly enhance the prospects for continued survival of this endemic primate and, combined with many other recent discoveries in the country, will contribute to the recognition of the archipelago as a globally significant biodiversity conservation priority [2], [5], [49].

## Supporting Information

Appendix S1 **Review of Philippine tarsier taxonomy.**
(PDF)

Appendix S2 **Vouchers, locality data, GPS coordinates, and Genbank numbers for included samples.**
(PDF)
