# Supplementary material for: Conservation Genetics of the Philippine Tarsier: Cryptic Genetic Variation Restructures Conservation Priorities for an Island Archipelago Primate
Source: PLoS One. 2014 Aug 19;9(8):e104340. doi: 10.1371/journal.pone.0104340 (PMC4138104; doi:10.1371/journal.pone.0104340)
Supplement: Appendix S1 — Review of Philippine tarsier taxonomy. (PDF) [file pone.0104340.s001.pdf]

Conservation genetics of the Philippine tarsier: cryptic genetic variation restructures conservation priorities in an island archipelago primate

Rafe M. Brown, Jennifer A. Weghorst, Karen V. Olson, Mariano R. M. Duya, Anthony J. Barley, Liza V. Duya, Myron Shekelle, Irene Neri-Arboleda, Jacob A. Esselstyn, Nathaniel J. Dominy, Perry S. Ong, Adrian Luczon, Gillian L. Moritz, Mae L. D. Diesmos, Arvin C. Diesmos, and Cameron D. Siler

Electronic Supplemental Material

Appendix S1

REVIEW OF PHILIPPINE TARSIER TAXONOMY

Previously proposed and current Philippine tarsier taxonomy has been based on external morphology, studied in preserved museum specimens. The distribution of available specimens is skewed toward large numbers collected from two sites on Mindanao Island, both of which fall within the range of *T. s. carbonarius* [1]. The first record of tarsiers in the literature is that of Pettiver, which in turn was based on a report by the Jesuit missionary G. J. Camel. This account became the basis for Linnaeus's [2] taxon *Simia syrichta*, a species without a type specimen, and for which the type locality was erroneously identified as Luzon. Cabrera [3] noted that tarsiers are absent on Luzon, and mentioned their presence on Samar. The description by Linnaeus is not sufficiently detailed to localize its provenience within the Philippines, and given the lack of a type specimen, subsequent taxonomists have used Samar as the type locality for "*Simia*" *syrichta*. Use of the genus *Tarsius* dates to Storr [4].

Modern tarsier taxonomy is based largely upon Hill [5], who classified tarsiers into one genus, of three species, with each species having three or more subspecies that are diagnosable with varying degrees of confidence. For *Tarsius syrichta*, Hill recognized three subspecies, each of which he considered poorly defined. He identified *T. syrichta syrichta* from Samar and Leyte, considered it a senior subjective synonym for Meyer's *T. philippensis* [6], and reclassified both *T. carbonarius* [7] and *T. fraterculus* [8] as subspecies of *T. syrichta*. The first of these was based upon specimens from the Gulf of Davao, on the southeastern side of Mindanao, and from the valley of the River "Poulangui" (=Pulangi, Pulangui) in Cotabato province. The second of these is based upon material from Sevilla, Bohol.

Since the time of Linnaeus's first scientific description of a tarsier, a stable taxonomy for tarsiers has been hindered by poorly defined or misidentified type localities, and difficult-to-locate or non-existent type specimens [1,9]. In the case of Philippine tarsiers the three poorly defined subspecies are as follows [5]:

1. *Tarsius syrichta syrichta* Linnaeus 1758
    type specimen: none

type locality: not Luzon, but Samar by convention

-syn: *Tarsius philippensis* Meyer 1897
type specimen: Dresden
type locality: Samar

2. *Tarsius syrichta fraterculus* Miller 1911
type specimen: possibly deposited in former Bureau of Science collections (likely destroyed in WWII)
type locality: Sevilla, Bohol

3. *Tarsius syrichta carbonarius* Heude 1898
type specimen: "presumably in Shanghai"
type locality: "*Golfe de Davao, et vallée du rio Poulangui, Mindanao*"

One other taxonomic consideration for Philippine tarsiers concerns their generic affiliation. For more than one hundred years, at least, all extant tarsiers have been classified within a single genus, *Tarsius*. Groves and Shekelle [10] recognized three genera of tarsiers, resuscitating Swainson's [11] *Cephalopachus* for the Sundaland tarsiers, and creating *Carlito* for Philippine tarsiers. This history of separation dates back at least as far as Musser and Dagosto [12], who examined morphology of museum specimens and identified a relationship between Sundaland and Philippine tarsiers, relative to the various Sulawesi tarsiers. This grouping was later supported with phylogenetic analysis of mtDNA sequence data, which showed robust support for a Sundaland-Philippine clade [13]. Groves [14], examining the morphological evidence, was the first to suggest a generic split between these clades, observing that the name *Rabienus* was available for the Sulawesi tarsiers. This initial step toward reclassifying *Tarsius* into multiple genera was incorrect on two counts, both of which Groves later corrected. First, long consideration of the provenience of Erxleben's [15] *Lemur spectrum* led to strong speculation that it was a Sulawesi tarsier, not a Philippine tarsier as Hill had assumed [1]. This was later confirmed with the rediscovery of the type specimen in the Paris Museum [8]. Thus it was the Sundaland-Philippine clade that required a new name (not the Sulawesi clade) if a three genera taxonomy was to be adopted. The senior generic name available for that clade was *Cephalopachus*, which Swainson [11] had used for *Tarsius bancanus* Horsfield 1828. Potentially relevant to the question of taxonomic partitioning of *Tarsius*, Meireles et al. [16] estimated a 5.6 Ma divergence between Philippine and Sundaland tarsiers. Under Goodman et al.'s [17] proposal to base high level classification on estimated divergence date, this divergence might warrant generic separation [10]. Subsequent estimates of the Sundaland-Philippine split have varied from 11.1 (4.8–18.6 Ma; [13]) to 30.8 (23.4–38.6 Ma; [18]). Some molecular systematists have adopted the three genera classification [19,20].

SUPPLEMENTAL REFERENCES

1. Groves C, Shekelle M, Brandon-Jones D (2008) Taxonomic history of the tarsiers: evidence for the origins of Buffon's tarsier, and the fate of *Tarsius spectrum* Pallas,

1778. In: Shekelle M, Maryanto I, Groves C, Schulze H, Fitch-Snyder H, editors. Primates of the oriental night. Jakarta: Indonesian Institute of Sciences (LIPI), LIPI Press. pp. 1-12.

2. Linnaeus C (1758) Systema naturae per regna tria naturae, secundum classes, ordines, genera, species, cum synonymis, locis. I. 10th ed. Stockholm: Laurent Salvi.
3. Cabrera A (1923) On the identification of *Simia syrichta* Linnaeus. J Mammal 4: 89-91.
4. Storr GLC (1780) Prodromus methodi Mammalium. Tubingen, Germany: Wolffer.
5. Hill WCO (1955) Primates: comparative anatomy and taxonomy. II. Haplorhini: Tarsioidea. Edinburgh: Edinburgh University Press.
6. Meyer AB (1897) Säugethiere vom Celebes- und Philippinen-Archipel, I. Abhandlungen und Berichte der Kaiserlich Zoologische und Anthropologische-Ethnologische Museum zu Dresden 6: 1-36.
7. Heude PM (1898) Etudes odontologiques: quatrièeme partie. Quadrumanes. Chap. I, Lémuriens, Tarsiens, Galéopitheciens et Cébiens. Mémoires concernant l'Histoire Naturelle de l'Empire Chinois 4: 155-208.
8. Callou C, Cuisin J, Groves C (2010) The rediscovery of Buffon's tarsier. Int J Primatol 31: 73-75.
9. Miller GS (1910) Descriptions of two new genera and sixteen new species of mammals from the Philippine Islands. P US Nat Mus 38: 391-404.
10. Groves C, Shekelle M (2010) The genera and species of Tarsiidae. Int J Primatol 31: 1071-1082.
11. Swainson W (1835) On the natural history and classification of quadrupeds. London, UK: Longman, Rees, Orme, Brown, Green, Longman and Taylor.
12. Musser GG, Dagosto M (1987) The identity of *Tarsius pumilus*, a pygmy species endemic to the montane mossy forests of Central Sulawesi. Am Mus Novit 2867: 1-53.
13. Shekelle M, Meier R, Wahyu I, Ting N (2010) Molecular phylogenetics and chronometrics of Tarsiidae based on 12S mtDNA haplotypes: evidence for Miocene origins of crown tarsiers and numerous species within the Sulawesian clade. Int J Primatol 31: 1083-1106.
14. Groves, CP (1998) Systematics of tarsiers and lorises. Primates 39: 13-27.
15. Erxleben JCP (1777) Systema regni animalis per classes, ordines, genera, species, varietates: cum synonymia et historia animalium. Impensis Weygandianis: Lipsiae.
16. Meireles CM, Czelusniak J, Page SL, Wildman DE, Goodman M (2003) Phylogenetic position of tarsiers within the order Primates: evidence from γ-globin DNA sequences. In: Wright PC, Simons EL, Gursky S, editors. Tarsiers: Past, present, and future. New Brunswick, NJ: Rutgers University Press. pp. 145-160.
17. Goodman M, Porter CA, Czelusniak J, Page SL, Schneider H et al. (1998) Toward a phylogenetic classification of primates based on DNA evidence complemented by fossil evidence. Mol Phylogenet Evol 9: 585-598.
18. Matsui A, Rakotondraparany F, Munechika I, Hasegawa M, Horai S (2009) Molecular phylogeny and evolution of prosimians based on complete sequences of mitochondrial DNAs. Gene 441: 53-66.
19. Springer MS, Meredith RW, Gatesy J, Emerling CA, Park J et al. (2012) Macroevolutionary dynamics and historical biogeography of primate diversification

inferred from a species supermatrix. PLoS ONE 7: e49521. doi:10.1371/journal.pone.0049521.
20. Finstermeier K, Zinner D, Brameier M, Meyer M, Kreuz E et al. (2013) A mitogenomic phylogeny of living primates. PLoS ONE 8: e69504. doi:10.1371/journal.pone.0069504.
