# Supplementary material for: Conservation Genetics of the Philippine Tarsier: Cryptic Genetic Variation Restructures Conservation Priorities for an Island Archipelago Primate
Source: PLoS One. 2014 Aug 19;9(8):e104340. doi: 10.1371/journal.pone.0104340 (PMC4138104; doi:10.1371/journal.pone.0104340)
Supplement: Appendix S2 — Vouchers, locality data, GPS coordinates, and Genbank numbers for included samples. (PDF) [file pone.0104340.s002.pdf]

Conservation genetics of the Philippine tarsier: cryptic genetic variation restructures conservation priorities in an island archipelago primate

Rafe M. Brown, Jennifer A. Weghorst, Karen V. Olson, Mariano R. M. Duya, Anthony J. Barley, Liza V. Duya, Myron Shekelle, Irene Neri-Arboleda, Jacob A. Esselstyn, Nathaniel J. Dominy, Perry S. Ong, Adrian Luczon, Gillian L. Moritz, Mae L. D. Diesmos, Arvin C. Diesmos, and Cameron D. Siler

Electronic Supplemental Material

Appendix S2

Vouchers, locality data, GPS coordinates, and GenBank numbers for included samples. All corresponding microsatellite data are deposited in Dryad (doi:10.5061/dryad.r7468).

**Sample ID**	**Island**	**Province**	**Municipality**	**Baragay**	**Local area name**	**Locality Code**	**Longitude**	**Latitude**	**Genbank (12S, CytB, ND2)**
AJ1/AI0001	Bohol	Bohol	Corella	Canapnapan		A	9.694533	123.927572	KM217270, KM217300, KM217324
AJ2/AI0002	Bohol	Bohol	Corella	Canapnapan		A	9.694533	123.927572	KM217271, KM217301, KM217325
AJ3/AI0003	Bohol	Bohol	Corella	Canapnapan		A	9.694533	123.927572	
AJ4/AI0004	Bohol	Bohol	Corella	Canapnapan		A	9.694533	123.927572	KM217272, KM217302, KM217326
AJ5/AI0005	Bohol	Bohol	Corella	Canapnapan		A	9.694533	123.927572	
AJ6/AI0006	Bohol	Bohol	Corella	Canapnapan		A	9.694533	123.927572	
AJ7/AI0007	Bohol	Bohol	Corella	Canapnapan		A	9.694533	123.927572	
AJ8/AI0008	Bohol	Bohol	Corella	Canapnapan		A	9.694533	123.927572	
AJ9/AI0009	Bohol	Bohol	Corella	Canapnapan		A	9.694533	123.927572	
AJ10/AI0010	Bohol	Bohol	Corella	Canapnapan		A	9.694533	123.927572	
AJ11/AI0011	Bohol	Bohol	Corella	Canapnapan		A	9.694533	123.927572	
AJ12/AI0012	Bohol	Bohol	Corella	Canapnapan		A	9.694533	123.927572	
AJ13/AI0013	Bohol	Bohol	Corella	Canapnapan		A	9.694533	123.927572	KM217273, KM217303, KM217327
AJ14/AI0001	Mindanao	Surigao Del Norte				B	9.448593	125.677050	
AJ15/AI0002	Mindanao	Surigao Del Norte				B	9.448593	125.677050	KM217295, ————, KM217349
AJ16/AI0003	Mindanao	Surigao Del Norte				B	9.448593	125.677050	
AJ17/AI0001	Bohol	Bohol	Corella	Canapnapan		A	9.694533	123.927572	
AJ18/AI0001	Bohol	Bohol	Corella	Canapnapan		A	9.694533	123.927572	
AJ19/AI0002	Bohol	Bohol	Corella	Canapnapan		A	9.694533	123.927572	
AJ20/AI0003	Bohol	Bohol	Corella	Canapnapan		A	9.694533	123.927572	
AJ21/AI0004	Bohol	Bohol	Corella	Canapnapan		A	9.694533	123.927572	

AJ22/AI0005	Bohol	Bohol	Corella	Canapnapan		A	9.694533	123.927572	
AJ23/AI0006	Bohol	Bohol	Corella	Canapnapan		A	9.694533	123.927572	KM217274, KM217304, KM217328
AJ24/AI0007	Bohol	Bohol	Corella	Canapnapan		A	9.694533	123.927572	
AJ25/AI0008	Bohol	Bohol	Corella	Canapnapan		A	9.694533	123.927572	
AJ26/AI0009	Bohol	Bohol	Corella	Canapnapan		A	9.694533	123.927572	
AJ27/AI0010	Bohol	Bohol	Corella	Canapnapan		A	9.694533	123.927572	
TG1/ 2008-001	Bohol	Bohol	Corella	Canapnapan		A	9.694533	123.927572	KM217275, KM217305, KM217329
TG2/ 2008-002	Bohol	Bohol	Corella	Canapnapan		A	9.694533	123.927572	KM217276, KM217306, KM217330
MVD254	Mindanao	Agusan Del Sur	Bunawan	Bunawan	San Marcos	C	8.222389	125.932444	KM217287, KM217317, KM217341
RMB10000	Mindanao	Zamboanga	Zamboanga City	Zamboanga	Pasonanca	D	6.977	122.067	KM217292, KM217322, KM217346
RMB 8966	Leyte	Northern Leyte	Baybay		ViSCA	E	10.875194	124.72435	KM217279, KM217309, KM217333
RMB 8967	Leyte	Northern Leyte	Baybay		ViSCA	E	10.875194	124.72435	KM217280, KM217310, KM217334
RMB 8968	Leyte	Northern Leyte	Baybay		ViSCA	E	10.875194	124.72435	KM217281, KM217311, KM217335
RMB 8969	Leyte	Northern Leyte	Baybay		ViSCA	E	10.875194	124.72435	KM217282, KM217312, KM217336
JAE 1856	Samar	Samar	Taft	San Rafel		F	11.8292	125.2766	
JAE 1857	Samar	Samar	Taft	San Rafel		F	11.8292	125.2766	
JAE 1873	Samar	Samar	Taft	San Rafel		G	11.4879	125.2766	
JAE 1909	Samar	Samar	Taft	San Rafel		F	11.8292	125.2766	
JAE 2065	Leyte	Leyte	Baybay City	Kilim	Sitio San Vicente, Tobod River	H	10.74136	124.84216	KM217278, KM217308, KM217332
JAE 2433	Mindanao	Agusan Del Sur	San Francisco	Bayugan 2	Mount Magdiwata	I	8.477361	125.988306	KM217285, KM217315, KM217339
JAE 2434	Mindanao	Agusan Del Sur	San Francisco	Bayugan 2	Mount Magdiwata	I	8.477361	125.988306	KM217286, KM217316, KM217340
JAE 2762	Mindanao	Zamboanga City	Zamboanga City	Pasonanca	Sitio Canucutan	J	6.990033	122.06745	KM217293, KM217323, KM217347
JAE 3436	Bohol	Bohol	Sierra Bullones	Danicop	Sitio Sigpit	K	9.76375	124.2791	KM217277, KM217307, KM217331
MVD677	Dinagat	Dinagat	Loredo	San Juan		L	10.350003	125.616669	KM217294, ————, KM217348
MVD678	Mindanao	Misamis Oriental	Gingoog City	Kamanikan		M	8.822997	125.097636	
MVD679	Mindanao	Misamis Oriental	Gingoog City	Kamanikan		M	8.822997	125.097636	KM217288, KM217318, KM217342
MVD "Giant"	Mindanao	Misamis Oriental	Gingoog City	Kamanikan		M	8.822997	125.097636	KM217289, KM217319, KM217343
T1/TarMoto	Mindanao	Surigao del Norte	Tubod	Motorpool		N	9.63333	125.55	
T2/TarMoto	Mindanao	Surigao del Norte	Tubod	Motorpool		N	9.63333	125.55	
T3/TarMoto	Mindanao	Surigao del Norte	Tubod	Motorpool		N	9.63333	125.55	
T4/TarMoto	Mindanao	Surigao del Norte	Tubod	Motorpool		N	9.63333	125.55	
T5/TarMoto	Mindanao	Surigao del Norte	Tubod	Motorpool		N	9.63333	125.55	KM217296, ————, ————
T6/TarMoto	Mindanao	Surigao del Norte	Tubod	Motorpool		N	9.63333	125.55	
T7/TarMoto	Mindanao	Surigao del Norte	Tubod	Motorpool		N	9.63333	125.55	KM217297, ————, ————
T8/TarMoto	Mindanao	Surigao del Norte	Tubod	Motorpool		N	9.63333	125.55	KM217298, ————, ————
T9/TarMoto	Mindanao	Surigao del Norte	Tubod	Motorpool		N	9.63333	125.55	KM217299, ————, ————
T10/TarMoto	Mindanao	Surigao del Norte	Tubod	Motorpool		N	9.63333	125.55	
T11/TarMoto	Mindanao	Surigao del Norte	Tubod	Motorpool		N	9.63333	125.55	
T12/TarMoto	Mindanao	Surigao del Norte	Tubod	Motorpool		N	9.63333	125.55	
MRMD1101	Mindanao	Sarangani	Kiamba	Bardtasan	Sitio Banate	O	5.997389	124.621697	KM217290, KM217320, KM217344
MRMD1102	Mindanao	Sarangani	Kiamba	Bardtasan	Sitio Banate	O	5.997389	124.621697	KM217291, KM217321, KM217345

MRMD1106	Mindanao	Surigao del Norte				–			
MRMD1107	Samar	Eastern Samar	Taft	San Rafel	Taft Forest	P	11.903092	125.418833	
MRMD1108	Samar	Eastern Samar	Taft	San Rafel	Taft Forest	P	11.903092	125.418833	KM217283, KM217313, KM217337
MRMD1109	Samar	Eastern Samar	Taft	San Rafel	Taft Forest	P	11.903092	125.418833	KM217284, KM217314, KM217338
JQ 630481	Mindanao	Agusan del Norte	Butuan City			Q	8.914427	125.507198	
JQ 630499	Mindanao	Agusan del Norte	Butuan City			Q	8.914427	125.507198	
JQ 630501	Mindanao	Agusan del Norte	Butuan City			Q	8.914427	125.507198	
